# Serious Illness Conversations in the Emergency Department for Older Adults With Advanced Illnesses: A Randomized Clinical Trial

**DOI:** 10.1001/jamanetworkopen.2025.16582

**Published:** 2025-06-18

**Authors:** Kei Ouchi, Susan D. Block, Dorene M. Rentz, Donna L. Berry, Hannah Oelschlager, Youkie Shiozawa, Sarah Rossmassler, Amanda L. Berger, Mohammad A. Hasdianda, Wei Wang, Edward Boyer, Rebecca L. Sudore, James A. Tulsky, Mara A. Schonberg

**Affiliations:** 1 Harvard Medical School, Boston, Massachusetts; 2 Department of Emergency Medicine, Brigham and Women’s Hospital, Boston, Massachusetts; 3 Department of Supportive Oncology, Dana-Farber Cancer Institute, Boston, Massachusetts; 4 Division of Palliative Medicine, Department of Medicine, Brigham and Women’s Hospital, Boston, Massachusetts; 5 Department of Neurology, Brigham and Women’s Hospital, Boston, Massachusetts; 6 Department of Biobehavioral Nursing and Health Informatics, University of Washington School of Nursing, Seattle; 7 Department of Nursing, MGH (Massachusetts General Hospital) Institute on Health Professions, Boston; 8 Division of Geriatrics and Palliative Care, Baystate Medical Center, Springfield, Massachusetts; 9 Division of Circadian and Sleep Disorders, Departments of Medicine and Neurology, Brigham and Women’s Hospital, Boston, Massachusetts; 10 Department of Emergency Medicine, The Ohio State University Wexner Medical Center, Columbus; 11 Division of Geriatrics, Department of Medicine, University of California, San Francisco; 12 Department of Medicine, Beth Israel Deaconess Medical Center, Boston, Massachusetts

## Abstract

**Importance:**

Conversations between seriously ill patients and clinicians about values and goals (ie, serious illness conversations [SICs]) can lead to patient-centered care toward the end of life. However, many patients have not had SICs when arriving in the emergency department (ED) and are at risk of receiving undesired care.

**Objective:**

To determine the effect of an ED-based, multimodal SIC intervention (known as ED GOAL) compared with usual care on patient-reported engagement in advance care planning (ACP) and clinician-documented SICs after leaving the ED.

**Design, Setting, and Participants:**

This 2-armed, 1:1, parallel-design randomized clinical trial was conducted at 3 participating EDs (2 academic medical centers and 1 community hospital) in Boston, Massachusetts, within a single health system between March 1, 2022, and July 1, 2024, with a follow-up duration of 6 months. Eligible participants were English-speaking adults 50 years or older with serious illnesses, including mild cognitive impairment or mild dementia, along with their caregivers. For patients with moderate to severe dementia, caregivers were the primary participants in the study. Patients with documented goals for medical care or physician orders in the last 3 months in medical records or deemed clinically inappropriate by the treating ED team were excluded.

**Intervention:**

SIC-trained research nurses conducted (1) a motivational interview about SICs, (2) a structured SIC, and (3) communication priming for the patients and their primary clinicians to reinitiate SICs on a tablet computer in the ED or within 1 week after leaving the ED.

**Main Outcomes and Measures:**

The primary outcome was a validated survey of patient-reported engagement in ACP at 1 month, with total possible scores ranging from 1 to 5, with higher scores indicating more engagement. Secondary outcomes included clinician documentation of end-of-life values and preferences in the medical record and completed advance directives.

**Results:**

A total of 141 patients (mean [SD] age, 66.7 [9.2] years; 73 [51.8%] female), predominantly diagnosed with metastatic cancer (85 [60.3%]), were enrolled and randomized to the intervention (n = 70) or usual care (n = 71). At 1 month, no difference was observed in patient-reported engagement in ACP (mean [SD] score, 3.32 [1.28] for control vs 3.37 [1.07] for intervention; maximum possible score, 5.00; *P* = .58), yet 12 patients in the intervention group (17.1%) discussed care preferences with their physicians compared with 5 (7.0%) control group patients (*P* = .07). Medical record documentation of end-of-life values and goals was significantly higher in the intervention group at 3 months (17 [24.3%] vs 7 [9.9%]; *P* = .03) and 6 months (22 [31.4%] vs 9 [12.7%]; *P* =  .008).

**Conclusions and Relevance:**

In this randomized clinical trial of seriously ill older adults in the ED, while a nurse-led SIC intervention did not significantly improve patient-reported engagement in ACP, it did increase clinician-documented SICs in the medical records. ED visits may serve as a critical access point to enhance SICs for seriously ill yet clinically stable older adults.

**Trial Registration:**

ClinicalTrials.gov Identifier: NCT05209880

## Key Points

**Question:**

Does an emergency department (ED)–based intervention to discuss serious illness care goals improve advance care planning outcomes for older adults with advanced illnesses?

**Findings:**

In this randomized clinical trial including 141 patients in the ED, a nurse-led intervention to discuss serious illness care goals did not significantly improve patient-reported engagement in advance care planning but did increase subsequent clinician-documented serious illness conversations in the medical records.

**Meaning:**

These findings suggest that ED visits may serve as a critical access point for serious illness conversations in clinically stable older adults with advanced illnesses.

## Teaser

This randomized clinical trial measures the effect of initiating conversations about serious illness care goals by trained nurses on patient- and caregiver-reported advance care planning engagement among older adults with advanced illness seen in the emergency department.

## Introduction

Serious illness conversations (SICs) are discussions between patients with serious, life-limiting illnesses (expected survival, <1 year) and clinicians that focus on the patients’ values and goals for care.^1^ These conversations promote shared decision-making and improved quality of life at the end of life.^2^ For seriously ill older adults, SICs are associated with lower rates of in-hospital death, less aggressive care at the end of life, earlier hospice referrals, increased peacefulness, and a greater likelihood of having end-of-life wishes followed.^2,3,4,5,6,7,8,9^ Clinician-documented SICs may reduce health care costs by 36%, potentially saving $1041 on average in the last week of life.^10^ Experts recognize that earlier SICs may “bend the cost curve” for health care,^11^ yet only 37% of seriously ill older adults have these conversations with their physicians,^2^ which occur on average 33 days before death.^12^

Emergency departments (EDs) are underutilized settings to engage seriously ill yet clinically stable older adults in SICs. About 75% of older adults visit the ED in the last 6 months of life,^13^ marking critical inflection points in their illness trajectories and signaling rapid decline.^14,15,16^ Although more than 70% of seriously ill patients express prioritizing comfort and quality of life over life extension,^17^ 56% to 99% lack advance directives available for use in the ED,^18^ putting them at risk of receiving care misaligned with their goals.^19^ To address this mismatch, we and other investigators^20,21,22^ developed and tested a behavioral intervention to engage seriously ill older adults in SICs (ED GOAL) to overcome the known barriers to SICs in this setting (eg, time constraints^23^). Based on social cognitive theory (ie, behaviors are learned from social contexts)^24^ and modeled from previously successful ED-based behavioral interventions^25,26,27,28,29,30^ using the transtheoretical model (ie, health behavior changes involve progress through different stages of readiness),^31^ ED GOAL is performed by trained research nurses speaking with the patients through videoconferencing (Zoom [Zoom Communications Inc]) in or after leaving the ED. ED GOAL consists of (1) a motivational interview for SICs, (2) a structured SIC using patient-tested language that has been studied rigorously in prior studies,^20,32,33^ and (3) a communication priming for the patient and their primary clinician to reintroduce SICs. ED GOAL has been demonstrated to be acceptable (N = 23)^21^ and feasible (N = 76)^34^ for seriously ill older adults in the ED. Although limited by the before-and-after design, investigators observed trends in increased frequency of new documentation of SICs by clinicians from 0 to 33%, health care proxy designation from 62% to 70%, and Medical Order for Life-Sustaining Treatment (MOLST) forms from 1% to 11% within 6 months after leaving the ED.^34^ Additionally, patient-reported engagement in advance care planning (ACP) improved significantly from 2.78 to 3.31 (*P* = .008) in the validated 4-item Advance Care Planning Engagement Survey (range, 1.00-5.00, with a higher score indicating better engagement,^35,36^ and 0.4 being a moderate effect size^37^).

Despite these promising findings from observational studies, the efficacy of ED GOAL had not been established in a randomized clinical study. Therefore, we conducted a randomized clinical trial of seriously ill older adults in 3 EDs within a single health system. The objectives were (1) to test the effect of ED GOAL administered by trained nurses on patient- and caregiver-reported ACP engagement after leaving the ED (primary outcome) and (2) to evaluate the impact of ED GOAL on patient-reported completion of SICs, newly documented SICs by clinicians in the medical records, patient-reported quality of communication, and survival.

## Methods

We conducted a 2-armed, 1:1, parallel-design, single-blind randomized clinical trial of seriously ill older adults in the ED. Massachusetts General Brigham’s Institutional Review Board approved all study procedures. The detailed study protocol has been previously published.^38^ The formal trial protocol is available in Supplement 1. All participants provided verbal informed consent. This study followed the Consolidated Standards of Reporting Trials (CONSORT) reporting guideline.

### Setting

All 3 participating EDs were in Boston, Massachusetts, and the study was conducted between March 1, 2022, and July 1, 2024. The 2 quaternary care academic medical centers were a 1059-bed hospital with 100 000 annual ED visits and a 793-bed hospital with 57 000 annual ED visits. The single community hospital had 171 beds and 30 000 annual ED visits. All 3 EDs provide clinical care 24 hours per day, 7 days per week.

### Participants

#### Inclusion Criteria

We included English-speaking adults, 50 years or older, with serious illnesses (metastatic cancer, oxygen-dependent chronic obstructive lung disease, chronic kidney disease receiving dialysis, New York Heart Association class III or IV heart failure, or treating ED clinician “would not be surprised if the patient died in the next 12 months,” which is known to be a factor associated with poor survival).^39,40^ Patients with nonmetastatic cancer, chronic obstructive lung disease not receiving home oxygen, chronic kidney disease not receiving dialysis, or New York Heart Association class I or II heart failure were also eligible if they were hospitalized in the last 12 months for their serious illness. Patients with mild cognitive impairment or mild dementia were eligible to participate with their caregiver as their study partner. For patients with moderate or severe dementia, the caregivers were the sole participants for the study. The caregivers were defined as health care proxies or next of kin.^38^ Sex and race and ethnicity (categorized as Asian, Black or African American, Hispanic or Latino, non-Hispanic or non-Latino, and White) were determined using self-reported data in the electronic health record. We included race and ethnicity in the analysis to explore the potential for differential effects.

#### Exclusion Criteria

Patients were excluded if they had documented goals for medical care in the last 3 months or MOLST in the medical records. We also excluded those determined to be clinically inappropriate by the treating ED or outpatient clinicians (eg, suicidal, diagnosed with a new serious illness in the ED, etc), with logistical challenges to enrollment, with delirium, who received both primary and specialty care outside of our health system, or had been enrolled in our previous feasibility study.

#### Recruitment

Our research team reviewed the medical records daily for potentially eligible patients and contacted them during their ED visit or within 10 days of discharge. When COVID-19 restrictions prevented us from physically conducting the intervention in the ED, we conducted our study virtually via a secure videoconferencing platform. Enrollment occurred in the ED, during a hospital stay, or after discharge. The study CONSORT diagram is shown in the Figure.

**Figure.**
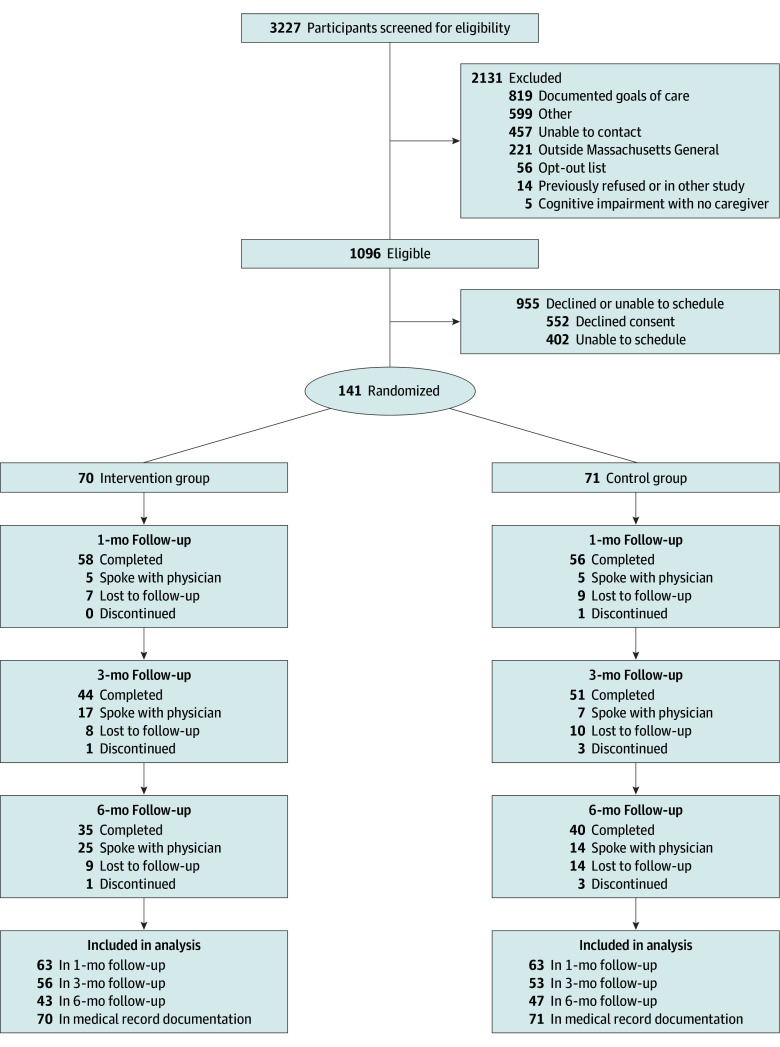
CONSORT Diagram

Study clinicians explained the study, obtained informed consent, and screened participants for delirium and cognitive impairment. Participants received a $48 gift card for participation. To comply with the National Institutes of Health recommendations for clinical trials, once we reached 50% of the enrollment target goals for each arm, we began to recruit minority and underrepresented populations preferentially. After reaching the quota for participants who self-identified as non-Hispanic White, we stopped recruiting these participants and only recruited minority and underrepresented populations.

### Intervention

#### Randomization and Blinding

We used an algorithm for computer-generated block randomization of 4. The allocation sequence and computer tool were generated by a biostatistician from our study team (W.W.). A research assistant (H.O. and Y.S.) then notified the study clinician privately of the allocation, thereby keeping the patient blinded to the treatment allocation. The blinded research assistants who performed the follow-up outcome assessments did not have access to the randomization tool or any baseline data that indicated the group to which the participant had been assigned. The biostatistician (W.W.) who conducted the data analysis was also blinded to the treatment allocation. It was not feasible to blind the study clinician to the treatment allocation.

#### Intervention and Control Arms

ED GOAL, described previously with high intervention fidelity,^21,22,41^ is a multimodal intervention performed by research nurses speaking with the patients (or caregivers if the patient is cognitively impaired) through a tablet computer through the videoconferencing platform in or after leaving the ED, consisting of (1) a motivational interview about ACP based on the social cognitive theory and transtheoretical model for health behavior change,^42^ (2) a structured SIC,^20,32,33^ and (3) a communication priming for the patient and the patient’s primary clinicians to discuss ACP.^43^ ED GOAL typically takes place once for approximately 15 minutes. The study team documented in the medical record what participants shared regarding their values and preferences for care in the event of advanced illness. The study team communicated these findings to patients’ outpatient clinicians with their permission and provided a handout designed to encourage further SICs between clinicians and families.^43^ The values and preferences categories discussed in the structured SIC used patient-tested language that has been studied rigorously in prior studies^20,32,33^ and also modified for individuals with cognitive impairment and their caregivers.^44^ The study team scheduled follow-up appointments with patients’ outpatient clinicians whenever feasible and desired by participants.

Intervention fidelity was measured for all enrollments to ensure that study clinicians delivered the intervention consistently. Concordant with expert guidelines, high intervention fidelity was defined as mean adherence to more than 70% of the components on the fidelity checklist.^41,45^ A doctoral-level nurse champion with a specialty-level certification in palliative care (S.R.) reviewed every patient enrollment using a previously developed intervention fidelity checklist (eAppendix in Supplement 2)^41^ and provided individualized and ongoing coaching to the trained research nurses throughout the study.

#### Usual Care

Control participants received usual care consisting of informing patients about the existence of health care proxy forms. Baseline and follow-up assessments were conducted similarly to those in the intervention arm.

### Outcome Measures 

Our primary outcome was the change in patient-reported engagement in ACP to discuss their values and preferences with outpatient clinicians (item 3 in the validated, 4-item Advance Care Planning Engagement Survey,^35,36^ “How ready are you to talk to your doctor about the kind of medical care you would want if you were very sick or near the end of life?” in a 5-point Likert scale, ranging from “I have never thought about it (1)” to “I have already done it (5)” 1 month after the ED visit. Our secondary outcomes were 3 additional items on the Advance Care Planning Engagement Survey to measure participants’ readiness to appoint a health care proxy, discuss goals of care with their health care proxy, and sign official documents to put their wishes in writing; new clinician documentation of SICs in medical records (not including the documentation by our study clinicians in the intervention group); self-reported occurrence of SICs by patients and/or caregivers evaluated with a previously validated dichotomous survey item^43,46^; the Heard and Understood Survey,^47^ modified to fit the context of SICs (“How well [do] they feel heard and understood by their primary outpatient clinician about medical care they would want if they were to get sicker?” on a 5-point Likert scale ranging from “not at all [1]” to “completely [5]”); and quality of communication survey^48^ (4 end-of-life items selected a priori and scored on an 11-point Likert scale ranging from “worst you can imagine [0]” to “best you can imagine [10]”) at 1, 3, and 6 months from baseline. We chose new clinician documentation of SICs in medical records as our secondary outcome because this provides definitive evidence of subsequent SICs following our intervention, which is our targeted behavior change. At the same time, this outcome seems challenging to obtain; thus, we selected patient-reported engagement in ACP as our primary outcome, recognizing that it serves as a surrogate marker of the targeted behavior change outcome.

Patient-reported outcomes were collected over the phone by trained research assistants before the intervention and at 1, 3, and 6 months after the intervention. For the outcomes in medical records, trained research assistants completed medical record abstraction using a codebook to search for new SIC documentation by patients’ primary clinicians and advance directives (eg, MOLST) after leaving the ED using standardized methods.^49^

### Sample Size

We expected that 10% of enrolled patients would die before completing all follow-ups. Prior nonrandomized studies demonstrated a mean patient-reported ACP engagement change from 3.8 to 4.3 on a 5.00-point scale, corresponding to a moderate effect size of 0.5.^37^ With a sample size of 70 patients per group, we would have 80% power to detect a medium effect size of 0.5 between the 2 groups using a 2-sided Fisher exact test (α = .05).

### Statistical Analysis

We performed intention-to-treat analyses using SAS, version 9.4 (SAS Institute Inc). The mean changes in self-reported ACP engagement were compared between study arms. Within arms, we used a 1-sample binomial exact test of proportions for categorical outcomes (eg, medical record documentation of SICs) and Wilcoxon signed rank test for ordinal outcomes (eg, Advance Care Planning Engagement Survey) at baseline and at 1, 3, and 6 months after the intervention. Two-tailed *P* < .05 was used for the significance threshold. For sensitivity analyses, we accounted for the hospitalized participants with a propensity to have more SIC documentation than those without, so we used logistic regression in subgroup analysis for SIC documentation. We also performed sensitivity analyses for the survey participant types (eg, patients vs caregivers).

We used the last observation carried forward, mean imputation, and worst outcome imputation to account for missing data. In addition, all available data were included in the mixed-effects models.

## Results

### Patient Characteristics 

Of the 3227 patients screened, 141 were enrolled (Figure and Table 1). The mean (SD) age of participants was 66.7 (9.2) years, with 73 participants (51.8%) identifying as female and 68 (48.2%) as male. A total of 6 participants (4.3%) identified as Asian; 30 (21.3%), Black or African American; 5 (3.5%), Hispanic or Latino; 136 (96.5%), non-Hispanic or non-Latino; 103 (73.0%), White; and 2 (1.4%) declined to answer. Most participants (85 [60.3%]) had metastatic cancer. Participants in the control arm were generally older and more likely to be non-Hispanic or non-Latino and White (self-reported on electronic health record). The 6-month mortality rates of the participants were similar in the intervention and control groups, at 4 (5.7%) in the intervention arm and 4 (5.6%) in the control arm.

**Table 1.** Participant Characteristics

Characteristic	Participant group, No. (%)^a^		
	Total (N = 141)	Control (n = 71)	Intervention (n = 70)
Age, y			
Mean (SD)	66.7 (9.2)	67.8 (9.6)	65.6 (8.7)
Median (range)	66.0 (50.0-92.0)	68.0 (50.0-92.0)	66.0 (50.0-85.0)
Sex			
Female	73 (51.8)	38 (53.5)	35 (50.0)
Male	68 (48.2)	33 (46.5)	35 (50.0)
Race (self-identified)			
Asian	6 (4.3)	3 (4.2)	3 (4.3)
Black or African American	30 (21.3)	15 (21.1)	15 (21.4)
White	103 (73.0)	53 (74.6)	50 (71.4)
Declined to answer	2 (1.4)	0	2 (2.9)
Ethnicity (self-identified)			
Hispanic or Latino	5 (3.5)	1 (1.4)	4 (5.7)
Non-Hispanic or non-Latino	136 (96.5)	70 (98.6)	66 (94.3)
Serious illness			
Solid tumor cancer with metastases or recent hospitalization	85 (60.3)	46 (64.8)	39 (55.7)
COPD receiving home oxygen therapy or recent hospitalization	8 (5.7)	5 (7.0)	3 (4.3)
CHF (NYHA Class III to IV) or recent hospitalization	29 (20.6)	13 (18.3)	16 (22.9)
CKD receiving dialysis or recent hospitalization	14 (9.9)	4 (5.6)	10 (14.3)
ED clinician would not be surprised if patient died in the next 12 mo	5 (3.5)	3 (4.2)	2 (2.9)
Location of enrollment			
In person	24 (17.0)	13 (18.3)	11 (15.7)
Virtual	117 (83.0)	58 (81.7)	59 (84.3)
6-mo Mortality	8 (5.7)	4 (5.6)	4 (5.7)

Abbreviations: CHF, congestive heart failure; CKD, chronic kidney disease; COPD, chronic obstructive pulmonary disease; ED, emergency department; NYHA, New York Heart Association.

^a^ Percentages have been rounded and may not total 100.

### Self-Reported Outcomes

#### Advance Care Planning Engagement Survey

At 1 month, no change was observed in item 3 on the Advance Care Planning Engagement Survey (primary outcome), with mean (SD) scores of 3.32 (1.28) for control participants and 3.37 (1.07) for intervention participants (*P* = .58). Across all 4 items, both control and intervention arms showed no statistically significant changes in mean scores (Table 2).

**Table 2.** Advance Care Planning Engagement Survey

Time	Participant group, mean (SD) score^a^							
	Item 1^b^		Item 2^c^		Item 3^d^		Item 4^e^	
	Control	Intervention	Control	Intervention	Control	Intervention	Control	Intervention
Baseline	4.65 (0.77)	4.45 (1.05)	4.14 (1.26)	4.17 (1.22)	2.87 (1.40)	2.84 (1.29)	3.24 (1.36)	3.08 (1.35)
1 mo	4.64 (0.83)	4.43 (0.98)	4.40 (1.08)	4.32 (1.07)	3.32 (1.28)	3.37 (1.07)	3.60 (1.21)	3.42 (1.24)
3 mo	4.68 (0.75)	4.43 (1.02)	4.48 (1.02)	4.31 (1.09)	3.55 (1.25)	3.56 (1.11)	3.76 (1.23)	3.39 (1.18)
6 mo	4.77 (0.73)	4.48 (1.01)	4.66 (0.81)	4.46 (0.98)	3.70 (1.23)	3.73 (1.13)	3.73 (1.25)	3.38 (1.18)

^a^ Scores range from 1.00 (never thought about it) to 5.00 (already done it).

^b^ “How ready are you to sign official papers naming a person or group of people to make medical decisions for you?”

^c^ “How ready are you to talk to your decision maker about the kind of medical care you would want if you were very sick or near the end of life?”

^d^ “How ready are you to talk to your doctor about the kind of medical care you would want if you were very sick or near the end of life?”

^e^ “How ready are you to sign official papers putting your wishes in writing about the kind of medical care you would want if you were very sick or near the end of life?”

#### Medical Record Documentation of SICs and Advance Directives 

At baseline, no documentation was observed in either group. By 1 month, documentation rates were still low, with health care proxy forms documented for 2 patients in both groups (2.9% for intervention and 2.8% for control; *P* > .99) and MOLST forms for 3 patients in both groups (4.3% for intervention and 4.2% for control; *P* > .99). However, a higher nonsignificant rate of clinician documentation of end-of-life values and preferences was seen in the intervention group (10 [14.3%]) compared with the control group (5 [7.0%]; *P* = .18) (Table 3).

**Table 3.** Medical Record Documentation of Serious Illness Conversations and Advance Directives

Time	Cumulative medical record documentation by participant group, No. (%)									
	Health care proxy form			Medical orders for life-sustaining treatment form			Clinician documentation of end-of-life values and preferences			
	Control (n = 71)	Intervention (n = 70)	*P* value	Control (n = 71)	Intervention (n = 70)	*P* value	Control (n = 71)	Intervention (n = 70)	*P* value
Baseline	0	0	NA	0	0	NA	0	0	NA
1 mo	2 (2.8)	2 (2.9)	>.99	3 (4.2)	3 (4.3)	>.99	5 (7.0)	10 (14.3)	.18
3 mo	3 (4.2)	8 (11.4)	.13	5 (7.0)	7 (10.0)	.56	7 (9.9)	17 (24.3)	.03
6 mo	4 (5.6)	10 (14.3)	.10	5 (7.0)	8 (11.4)	.40	9 (12.7)	22 (31.4)	.008

Abbreviation: NA, not applicable.

At the 3-month follow-up, the intervention group showed a pattern of higher documentation, with health care proxy forms completed for 8 participants (11.4%) compared with 3 (4.2%) in the control group (*P* = .13). Clinician documentation of serious illness care goals was significantly higher in the intervention group (17 [24.3%]) compared with the control group (7 [9.9%]) (*P* = .03). MOLST form documentation remained similar between groups (7 [10.0%] and 5 [7.0%]; *P* = .56).

At 6 months, documentation for health care proxy forms continued to demonstrate nonstatistically significant increases in the intervention group (10 [14.3%] vs 4 [5.6%]; *P* = .10), while MOLST form documentation remained the same (8 [11.4%] and 5 [7.0%]; *P* = .40). Clinician documentation of end-of-life values and preferences was significantly higher in the intervention group (22 [31.4%]) compared with the control group (9 [12.7%]) (*P* = .008).

#### Self-Reported SICs With Primary Clinicians After Leaving the ED

At the 1-month follow-up, more patients in the intervention group (12 [17.1%]) reported having discussed with their primary physician the kind of medical care they would want if they became too sick to speak for themselves compared with the control group (5 [7.0%]), although there were no statistically significant differences (*P* = .07). Additionally, 33 patients in the intervention group (47.1%) indicated they had talked to their loved ones about their medical care preferences in the event they were too sick to speak for themselves compared with 28 (39.4%) in the control group, with no significant difference between the groups (*P* = .24).

#### Heard and Understood

At baseline, patients reported feeling significantly more “heard and understood” by their study clinicians who conducted ED GOAL compared with their primary physician (53 [75.7%] completely by study clinicians vs 30 [42.9%] completely by their primary physician; *P* < .008). These findings highlight a potentially significant gap in perceived communication and understanding between patients and their primary physicians vs their study clinicians. The perceived positive effects of the intervention could also explain this.

#### Quality of Communication

Overall, the 2 groups had no statistically significant differences for any of the items measured. However, the intervention group had a higher mean (SD) score when asked if their physicians inquired about the things in life that are important to them (8.5 [1.7] vs 7.8 [2.6]), although this difference was also not statistically significant (*P* = .34).

## Discussion

In this randomized clinical study, our ED-based, multimodal SIC intervention did not result in increased patient-reported engagement in ACP, but a notable increase (24.3% in the intervention arm vs 9.9% in the control arm; *P* = .03) was observed in the documentation of SICs in the medical record at 3 months. Furthermore, no statistically significant difference was observed in the self-reported SICs. The discrepancy between self-reported engagement and documented SICs may stem from many factors. We tried to maximize the chances of success in participant engagement by including a patient-facing handout modeled after a previously successful intervention.^33,43^ However, ED visits often represent inflection points in patients’ illness trajectories, usually followed by additional transitions such as hospitalization or rehabilitation, which may leave patients physically and psychosocially overwhelmed. In fact, many patients lowered their score from 5.00 (highest) to 3.00 (lower) on realizing, through our intervention, that they had not previously engaged in ACP. A well-known psychological phenomenon exists where, after receiving education about a specific task, individuals realize that they have not engaged in behaviors they had thought they completed, as seen in prior studies using the Advance Care Planning Engagement Survey.^50,51^ Discussing participants’ values and the possibility of getting sicker in the future may have given participants new insight into what engaging in ACP entails.

Despite the null results in patient-reported engagement in ACP, we found a statistically significant improvement in documentation of SICs between patients and clinicians. This improvement is a clinically meaningful change, similar in magnitude to high-quality ACP trials in other settings.^52,53^ The ultimate objective of our intervention was to empower patients to engage in SICs with their outpatient clinicians. Therefore, regardless of patients’ self-reported engagement, our findings indicate that the targeted behavior of SIC occurrence is changing. Even if patients did not report engagement, the documented conversations by their clinicians are clinically meaningful, as this is a more distal surrogate outcome. Given these findings, we will likely focus our primary outcome on the documentation of SICs in future studies.

Our study findings have several implications for researchers and clinicians. For researchers, we demonstrated that recruitment of seriously ill older adults during and shortly after acute care visits to engage them in SICs was feasible. By recruiting these patients around ED visits, we engaged them at clinically meaningful times in their illness trajectories. We also added care coordination components to communicate the values and preferences that our participants shared with their primary outpatient clinicians. For clinicians, interventions aimed at improving SICs between patients and clinicians have been demonstrated in randomized trials in other settings.^20,43,54^ These interventions lead to more frequent, earlier, and better SICs and to greater documentation of SICs in the medical records,^20,43^ resulting in reduced anxiety and depression in these patients.^54^ Patients who receive these interventions may also have lower health care costs.^55^ To our knowledge, this is the first randomized clinical trial to evaluate the efficacy of similar SIC interventions in ED settings, suggesting that ED clinicians, policy makers, and health insurers should consider ED visit–triggered SICs as clinically meaningful investment for seriously ill older adults. Much like nurse navigators from insurers who currently triage patients for acute care on the phone, offering early SICs for seriously ill older adults would be in the best interest of the patients and the insurers.

### Limitations

There are several limitations in this study. Due to COVID-19 restrictions, the intervention was adapted to a virtual format to continue the study. This change in intervention could have introduced an unmeasurable bias with uncertain effects on the outcomes. A block randomization was chosen to balance between the groups, which could have introduced selection bias. We were unable to determine the quality and fidelity of SICs conducted by inpatient or outpatient clinicians from the electronic health record. The study took place at a single health system in Boston, and only patients who were willing to enroll in a study were included, which may have biased the results toward the null (ie, patients who are willing to participate may be more engaged with SICs now and subsequently). In the future, we plan to collect the baseline self-efficacy in SICs and the prognostic awareness of participants to better understand how these factors may affect the results. Although ED practices are relatively standardized across the US, further research is needed to improve generalizability. Finally, our recruitment rates for minoritized groups were lower than other groups; therefore, future studies should prioritize the inclusion of more diverse patient populations to broaden the relevance of our findings.

## Conclusions

In this randomized clinical trial, a nurse-led, ED-based SIC intervention did not significantly improve patient-reported engagement in ACP. At the same time, it did increase clinician-documented SICs in the medical records. ED visits may serve as a critical access point to enhance SICs for seriously ill yet clinically stable older adults.

## Notes

Supplement 1.
Trial Protocol

Supplement 2. **eAppendix.** ED GOAL Intervention Script

Supplement 3.
Data Sharing Statement
