# Supplementary material for: Serious Illness Conversations in the Emergency Department for Older Adults With Advanced Illnesses: A Randomized Clinical Trial
Source: JAMA Netw Open. 2025 Jun 18;8(6):e2516582. doi: 10.1001/jamanetworkopen.2025.16582 (PMC12177648; doi:10.1001/jamanetworkopen.2025.16582)
Supplement: Supplement 1. — Trial Protocol [file jamanetwopen-e2516582-s001.pdf]

Mass General Brigham

Institutional Review Board
Intervention/Interaction Detailed Protocol

Principal Investigator: Kei Ouchi, MD, MPH

Project Title: An advance care planning intervention in the emergency department: a randomized controlled trial

Version Date: 2023.05.26

For Intervention/Interaction studies, submit a Detailed Protocol that includes the following sections. If information in a particular section is not applicable, omit and include the other relevant information.

1. Background and Significance

- **Summarize relevant literature, data, and historical background**

Advance care planning (ACP) conversations can lead to well-informed shared decision making and improved quality of life at the end of life.[1] For seriously ill older adults, ACP conversations are associated with lower rates of in-hospital death, less aggressive medical care at the end of life, earlier hospice referrals, increased peacefulness, and a 56% greater likelihood to have end-of-life wishes known and followed.[1-8] Furthermore, patients with documented ACP conversations experience a 36% reduction in the cost of end-of-life care, with an average cost savings of $1,041 per patient in the last week of life.[9] Experts recognize that earlier ACP conversations are the key to "bend the cost curve" for health care.[10] *Yet only 37% of seriously ill older adults have ACP conversations with their physicians,*[1] *on average 33 days before death.*[11]

Emergency departments serve as an ideal setting to engage seriously ill yet clinically stable older adults who may benefit from ACP conversations. During the last six months of life, 75% of older adults visit the emergency department (ED).[12] ED visits are inflection points in these patients' illness trajectories, signaling a more rapid rate of decline.[13-15] More than 70% of these patients express priorities focused on comfort and quality of life rather than life extension,[16] yet a systematic review revealed that 56% to 99% do not possess advance directives in the ED,[17] and many are at risk of receiving care that does not align with their goals.[18] *We propose to seize the opportunity of the ED visit to engage seriously ill older adults to initiate/reintroduce ACP conversations.*

ED clinicians lack practical methods to engage seriously ill older adults to engage in ACP conversations. ED clinicians recognize that an ED visit provides a time and location for seriously ill older adults to discuss ACP;[19] however, the time-pressured ED environment discourages clinicians from conducting in-depth conversations.[20] *Practical methods to overcome these ED-specific barriers are needed to engage patients who may benefit from ACP conversations*.

- **Describe previous pre-clinical or clinical studies leading up to and supporting the proposed research**

In our prior research, we developed and piloted a ED clinician-led, behavioral intervention (*ED GOAL*) designed to engage seriously ill yet clinically stable older adults in the ED to address ACP with their outpatient clinicians.[21,22] In an acceptability study, 82% (19/23 patients) found it acceptable and stated that it helped them engage in talking about their goals for future care with their outpatient clinicians.[21] In a feasibility study (N=50), average patient-reported ACP self-efficacy increased from

3.8 to 4.3 on a 5-point scale, new electronic medical record (EMR) documentation of ACP (new/change in health care proxy or advance directive forms) was found in 26% (13/50 patients), and new EMR documentation of ACP conversation (discussion of code status, health care proxy, or advance directive forms) was found in 40% (20/50 patients) of seriously ill older adults who underwent *ED GOAL* (under peer review). When COVID restrictions prevented us from conducting *ED GOAL* physically in the ED, we have demonstrated the feasibility of conducting our study virtually using phone/zoom (IRB Protocol 2020P002934, in preparation for publication).

- **Describe rationale behind the proposed research and significance to patients, society, and/or science**

Since the improvements in ACP engagement were seen in a one-arm, pre-/post-intervention feasibility study, we propose to test this in a randomized clinical trial (RCT) as a next step. *ED GOAL* will facilitate ACP conversations for seriously ill older adults in or after leaving the ED. These findings will be used for a future R01 proposal to definitively test the efficacy of *ED GOAL* in a large multi-site pragmatic trial. We aim to establish *ED GOAL* as a national standard of care to help seriously ill older adults to receive care concordant with their goals.

2. Specific Aims and Objectives

- **Specify objectives and hypotheses to be tested in the research project**

Aim 1: Test, in a randomized controlled design (N=180, 90 in each group), the effect of *ED GOAL* administered by trained nurses on patient and caregiver ACP engagement one month after leaving the ED.

Hypotheses: ACP engagement (a composite outcome combining patient-reported and EMR outcomes) will significantly increase in those who received *ED GOAL* compared to usual care.

Aim 2: Identify the patient-perceived benefits and obstacles of ACP conversations after *ED GOAL* using semi-structured interviews (N~30).

Rationale: Most patients who underwent our intervention in prior studies expressed their gratitude and reported how helpful the intervention was in their lives. Yet, these perceived benefits ("opened my eyes about what I need to focus in my life") may be difficult to capture using validated, quantitative instruments. Furthermore, we will also elicit patient-perceived barriers to completing the ACP conversations.

3. General Description of Study Design

- **Explain the basic study design, e.g., parallel group, randomized controlled trial, open-label single arm study, cross over, adaptive, etc.**

This is a two-armed, parallel-design, pre-/post-intervention assessment study. We will conduct a randomized controlled trial for *ED GOAL* on a cohort of 180 older adults with serious illness to collect patient-centered outcomes and determine preliminary efficacy on increasing ACP engagement (self-reported and/or in the electronic medical record) one month after leaving the ED. We will also conduct qualitative interviews with participants of *ED GOAL*.

- **Provide study schema as applicable (a schema is required for greater than minimal risk studies, and optional for minimal risk studies**

Figure 1 Study Schema

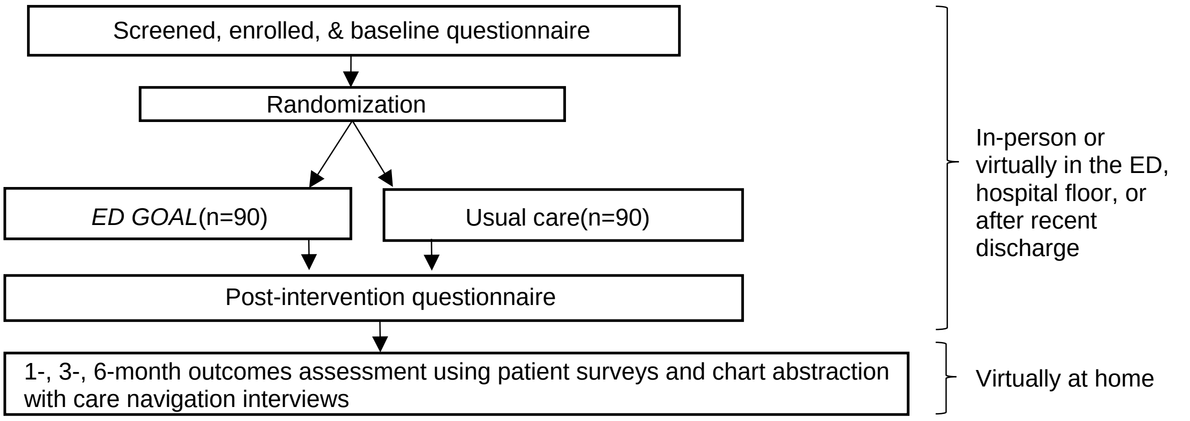

4. Subject Selection

Describe sources of subjects and procedures for subject selection, including the following:

- **Inclusion/Exclusion Criteria**

Participants will only consist of patients who meet the inclusion/exclusion criteria in **Table 1**. Serious Illness criteria will be determined by reviewing the medical record of potential participants for relevant diagnoses. Those with dementia or cognitive impairment will undergo a screening process using the QDRS, as described in Section 5. For eligible patients who have mild cognitive impairment (MCI) or mild dementia (as shown by cognitive impairment assessment), both the patient and the patient's caregiver will be enrolled as subjects in the study. For patients with moderate to severe dementia, the caregivers will be the study participants, and no involvement of the patients will occur. We will determine the potential subject's cognitive status by review of medical records and screening described in the "Subject Enrollment" section below.

The caregivers are defined as health care proxy or next of kin (when health care proxy has not been appointed). Patients without competency who has court-appointed legal guardians are excluded from this study given the nature of ACP conversations for this group is largely different from ordinary patient/caregivers. Given that this is a minimal risk study (i.e., patients and caregivers are routinely experiencing ACP conversations in their clinical encounters), we will obtain verbal consent/assent using the study information sheet.

Table 1 Patient eligibility criteria

Inclusion	Exclusion
1. ≥50 years of age AND ≥1 Serious illness*<br>*OR*<br>ED clinician would not be surprised if patient died in the next 12 months (a validated prognostic sign)[23,24]<br>2. English- or Spanish- speaking<br>3. Capacity to consent<br>a. Patient with mild cognitive impairment or mild dementia with capacity to consent (requires a caregiver/study partner to enroll)	1. Acute physical or emotional distress<br>2. Determined by treating or study clinician not to be appropriate<br>3. Clearly documented goals for medical care**<br>(Unless the treating or study clinician recommends that the intervention is clinically indicated)<br>4. Delirium (assessed using 3D-CAM if enrolled in ED/hospital)<br>5. Already enrolled in this study

b. Caregiver of patient with moderate/severe dementia with capacity to consent	6. Unable/unwilling to schedule the follow-ups on the calendar<br>7. Receive both the outpatient care for serious illness and primary care outside of the MGB system
*NYHA Stage III/IV congestive heart failure, chronic obstructive lung disease on home oxygen, chronic kidney disease on dialysis, metastatic solid tumor cancer, or dementia and cognitive impairment. In addition, patients with NYHA Stage I/II congestive heart failure, chronic obstructive lung disease not on home oxygen, chronic kidney disease not on dialysis will be included if recent hospitalization in the last 12 months exists.	**e.g., MOLST, medical order for life-sustaining treatment, documented serious illness conversations in clinician notes within the last 3 months, etc.

- **Local Recruitment Procedures:**
  Explain in detail the methods and procedures you will use to recruit participants. Describe in a step-by-step procedure below:

  - **How individuals are identified for recruitment including description of use of recruitment materials such as flyers, brochures, advertisements, letters, etc.**
    Our research team will review the medical records in Epic to identify the potential subjects to approach in this study. To identify potential subjects in the ED, we will review the ED patient lists Monday to Friday between 8am to 6pm. To capture potential subjects we missed during this screening period, we will also review medical records of patients who visited the ED in the last 10 days so that we can enroll them virtually after leaving the ED/hospital (if admitted).

  - **Who is responsible (role on research team) for identifying and recruiting individuals**
    Trained RAs will search through the medical records to find potential subjects meeting our inclusion and exclusion criteria. The study clinician consenting the subjects will double check the eligibility criteria prior to consent.

  - **When individuals are recruited**
    Subjects will be approached for recruitment at the time of ED visit, during their hospitalization after the ED visit, or within 10 days of leaving the ED.

  - **Where individuals are recruited**
    We will recruit subjects in the ED, ED observation unit, or hospital floor at Brigham and Women's Hospital, Massachusetts General Hospital, Brigham and Women's Faulkner Hospital, and Salem Hospital.
    We will make every attempt to enroll/approach the patient in the ED. We will follow the following steps:
    1. When potential subjects are identified in the ED, clinical appropriateness is assessed with the treating clinicians;
    2. The treating clinicians will introduce the study and obtain permission for our research team to approach the subject in the ED; and
    3. The study team will contact the patient for eligibility assessment and consent (if eligible).

    When in-person enrollment is not feasible due to COVID restrictions or logistical reasons (e.g., potential subjects are identified overnight when research team is not present), we will use the Patient Gateway recruitment strategy recommended by the IRB.

- **How recruitment goals match the prevalence rates of the condition/disease being studied and the populations most impacted by the condition/disease being studied**
  Our recruitment goals match the target populations of clinicians who routinely conduct ACP conversations in their clinical practice.

- **Methods to enhance enrollment of diverse individuals and under-represented populations**
  To comply with the NIH recommendations for clinical trials, once we approach 50% of enrollment target goals for each arm, we will begin to preferentially recruit minority and under-represented populations if they are <20% of the enrolled subjects to ensure that our study adequately represent the study population.

5. Subject Enrollment

- **Describe any pre-screening procedures as applicable. Indicate whether subjects will be prescreened over the phone and/or will be asked to provide separate informed consent specific to screening procedures.**

No pre-screening procedures are planned.

- **Describe in a step-by-step procedure the consent process including:**
  - **When and where informed consent will be obtained (including description of any electronic consenting procedures)**

    Timing of consent:
    The informed consent will be obtained at the time of ED visit, during the hospitalization, or after the potential subjects leave the ED in-person or over the phone/zoom. All study procedures including the consent process may occur in-person or over the phone/zoom depending on logistical restrictions (e.g., COVID surge restrictions) and patient/clinician preference.

    Sound amplification device (if feasible):
    Prior to the screening assessments, the study staff will offer a personal sound amplifier as a hearing-assistive device to subjects who may benefit from its use (e.g., subjects with hearing impairment issue). From our experience, given that the setting may be in the noisy ED, the subjects can experience difficulties answering our questions. Therefore, the hearing aids will help them respond to our prompts/instructions more appropriately.

    Subject identification:
    Trained RAs will use Epic to identify patients in the ED or the hospital 8am to 6pm Monday through Friday. To capture potential subjects that we may have missed, we will also review recent discharge list who might be eligible based on inclusion/exclusion criteria. Once a potential subject is identified, the trained RA will approach the treating clinician to see if the patient is eligible and have capacity to consent (if the potential subject is in the ED or in the hospital). The study procedure will pause whenever the study team is interrupted by clinical activities of the participants and resume when it is determined to be feasible again by the clinical team.

    Enrollment procedures and cognitive impairment screening:

Our trained study clinician will review the eligibility criteria with the RA prior to engaging with the potential subject. If the potential subject is agreeable to learn more about our study verbally, the study clinician will administer a validated, delirium screening (3D-CAM) if the patient is enrolled in the ED/hospital.[25] If the patient is found to have no delirium, the study clinician will classify the patients based on known diagnosis of cognitive impairment or by administering a validated, cognitive impairment screening (MiniCog and/or Quick Dementia Rating System, QDRS).[26,27] Depending on the cognitive impairment status, the patients and/or caregivers will be enrolled in the study (**Table 2**).

Table 2 Intervention target and outcome assessment

Cognition status	Study participants	Outcome assessment
Normal (MiniCog >2 and/or QDRS 0-1)	Patient only	ACP engagement for patient + EMR
Mild cognitive impairment and mild dementia (MiniCog<3 and QDRS 2-12)	Patient and caregiver	ACP engagement for patient + ACP engagement for caregiver + EMR
Moderate and advanced dementia (MiniCog<3 and QDRS 13-30)	Caregiver only	ACP engagement for caregiver + EMR

Upon classification of patients by their cognitive status, informed consent will be obtained using a 4-item consent capacity checklist (see attached 4-item consent capacity checklist). For patients with known/documented moderate/severe dementia per medical record or self-report, we will approach the caregiver for enrollment and perform the 4-point consent capacity checklist. If the caregiver states that the patient can assent (e.g., verbal, able to comprehend, etc.), we will obtain an assent from the patient. Patients are not the subjects of this study.

At this time, the study clinician will announce to the subject that the research procedure will begin.

- **A separate description for adults and children if applicable**
  Not applicable.

- **The process for obtaining consent from non-English speakers if applicable**
  Consent will be obtained by an English-speaking clinician and a hospital-certified interpreter.

- **The process to determine capacity to consent and use surrogate decision makers if applicable**
  We will conduct the following procedures to determine the capacity to consent:

  For subjects with <u>no</u> known diagnosis of <u>mild cognitive impairment or dementia</u> on medical record, we will ask the treating clinicians (if subjects are in the ED or in the hospital) regarding their assessment of capacity to consent and clinical stability to approach the subject. Then, the study clinician will follow the procedures described above to classify the patients based on cognitive impairment status and use the 4-item capacity checklist for consent.

  For subjects with a <u>known</u> diagnosis of <u>mild cognitive impairment or mild dementia</u>, we will ask the treating clinicians (if subjects are in the ED or in the hospital) regarding their

assessment of capacity to consent and clinical stability to approach the subject. At the same time, we will confirm the availability of a caregiver to serve as a study partner. If caregiver is unavailable, the subject will be excluded. Then, the study clinician will follow the procedures described above to classify the patients based on cognitive impairment status and use the 4-item capacity checklist for consent.

For subjects with a known diagnosis of moderate to advanced dementia, the caregivers will be consented for the study (patients will not be the subjects of our study). We will use the 4-item consent capacity checklist.

- **Procedures to minimize undue influence to enroll, particularly if recruiting the investigators' own patients**
  The study team will have no patient/clinician relationship with the potential subjects being approached in this study.

- **Describe post-consent intervention assignment and randomization method if applicable**

We will have three groups of participants based on cognitive ability for this study:
1) Patients with normal cognition
2) Patients with MCI or mild dementia + their caregivers (both are participants)
3) Caregivers of patients with moderate and advanced dementia (only the caregivers are the participants)

Upon classification of eligible participants into the correct group above, we will randomly assign them into one of two arms of the study using block randomization of four using a computer-generated algorithm:

1) Intervention (*ED GOAL*)
2) Control (usual care)

6. STUDY PROCEDURES

Provide detailed description of all study visits, procedures, and data collections, including:

- **Description of each study visit and procedures at each visit (include a schedule/table of study procedures)**

Initial enrollment (~30 to 60 minutes total):
After the consent, our trained study clinician will administer the intervention to the participant in-person, on the phone, or over zoom depending on participant preference. After the intervention is completed, the RA will obtain post-enrollment questionnaires and also schedule dates/times for the follow-up appointments. With participants' permission, the study clinician will document the clinically important findings elicited from the participant (e.g., preferences for end-of-life care goals) in Epic's ACP module and also send an email regarding the findings to patient's outpatient clinicians within Mass General Brigham health system via secure email. The study team will also provide the post-enrollment patient handout (see attachment) to the participants. All participants in the intervention arm will receive the same intervention and follow-up procedures.

Follow-up appointments (~30 to 45 minutes each):
During the scheduled follow-up appointments on the phone/zoom, our RA will administer surveys to assess for the outcomes. In addition, if the participants report that they have not had conversations with their outpatient clinicians about ACP, our study clinician will conduct brief coaching to

overcome the obstacles participants might be facing in this process and also document clinically relevant information in Epic to notify to the outpatient clinician.

**Table 3 Study visits**		
**Study visit**	**Intervention**	**Outcome assessment**
Initial enrollment	*ED GOAL*	Post-enrollment survey
1-month follow-up	ACP navigation coaching (as needed)	Follow-up patient-reported outcomes surveys
3-month follow-up	ACP navigation coaching (as needed)	Follow-up patient-reported outcomes surveys
6-month follow-up	ACP navigation coaching (as needed)	Follow-up patient-reported outcomes surveys

- **Description of study drugs, devices, or other interventions/exposures administered, including:**
  - **Dose, method of administration, schedule of administration, dose modifications**
    Our intervention is followed by scheduled, follow-up visits at one, three, and six months afterwards. The follow-up visits will be performed to assess the outcomes as well as to serve as the ACP care navigation/coaching by our study clinician.

    Study clinician training:
    We will train the study clinicians to administer these tools by 3-hour didactic on the concepts of delirium, cognitive impairment, and dementia, as well as bedside practice administration with the PI until competency is demonstrated (PI will self-record the responses next to the study clinician administering the instrument and compare the results. We will repeat this process with new patient volunteers until there is no discrepancy). In the case of equivocal findings on these assessments administered by study clinician, the PI backup will be available.

    Intervention fidelity:
    To ensure that all study clinicians deliver our intervention consistently, we will check the intervention fidelity. The percent adherence to the intervention fidelity checklist will be measured for every participant encounter (see attached fidelity checklist). High fidelity will be defined as mean adherence of >70% to the components on the fidelity checklist, which is consistent with expert consensus for behavioral intervention studies.[28]

  - **Justification and safety information if FDA approved drugs will be administered for non-FDA approved indications ("off-label"): How are doses, routes of administration, or participant populations different from FDA approved indicated use?**
    Not applicable.

- **Description of specific data variables to be collected, including data collection methods, assessments, data collection sheets, and/or schedule of assessments (A schedule/table of study assessments is preferred)**

**Table 4 Outcomes**		
**Measures**	**Descriptions**	**Timing**
ACP engagement survey for patients (primary outcome)	Used for patients, this validated instrument will measure the self-reported processes for ACP (knowledge, contemplation, self-efficacy, and readiness), and ACP behavior.[29]	Baseline & at 1, 3, and 6 months
ACP engagement survey for caregivers (primary outcome)	Used for surrogate decision-makers, this ACP engagement survey is validated to measure the same ACP processes and behaviors.[30]	Baseline & at 1, 3, and 6 months
EMR documentation	Review EMR to see if new documentation of ACP conversation,	Baseline & at 1, 6, and

(primary outcome)	change in code status, or advance directive forms documented by physicians or mid-level providers exist.	12 months
Self-reported ACP conversation survey (primary outcome)	Quantitative and qualitative questionnaires to assess the self-reported completion of ACP conversations with outpatient clinicians and families as well as barriers/facilitators of this process.	At 1, 3, and 6 months
Heard and understood survey (secondary outcome)	A validated instrument for seriously ill patients to report how well they feel heard and understood about their wishes for end-of-life care.[31]	Baseline & 1, 3, and 6 months
Quality of communication survey (secondary outcome)	A validated instrument to measure the quality of communication about end-of-life care.[32]	Baseline & at 1, 3, and 6 months
Healthcare utilization (secondary outcomes)	Review EMR to see # of urgent care visits, ED visits, hospitalizations, hospice visits, and outpatient visits.	At 6 and 12 months before and 1, 6, 12 months after enrollment
Mortality	Review EMR to determine patient's vital status.	At 1, 3, and 6 months
Qualitative benefits and obstacles of ACP conversations after *ED GOAL* (secondary outcome)	Semi-structured interviews to assess the benefits of *ED GOAL* and obstacles participants faced in completing more ACP conversations with their outpatient clinicians and loved ones after *ED GOAL* (see interview guide).	At 1, 3, and/or 6 months

- **Description of planned genetic research as applicable (e.g., specific description of whole genome sequencing, creation of immortalized cell lines or induced pluripotent stems cells, and sharing of genetic material with collaborators and central repositories as applicable, etc.)**
  Not applicable.

- **Description of plans for return of research results as applicable (e.g., specific description of, rationale for, and process by which research results will be returned, including to whom, by whom, and when, etc.). Include plans for managing incidental findings as applicable.**
  Not applicable.

- **Definition of primary and secondary outcomes/endpoints. Note that outcome measures should be quantifiable and measurable.**
  Please see **Table 4** above.

- **Definition of study termination criteria, e.g., objective criteria for clinical worsening, lack of improvement, and/or unacceptable adverse events**
  - We do not anticipate any adverse events (AE) for this study other than loss of confidentiality. Any AE will be reported to our institutional review board at the time of annual continuing review by the principal investigator using an Adverse Event Form in accordance with the MGB Human Research Committee guidelines for AE reporting.

- **Local site restrictions or site-specific procedures as applicable, including:**
  - **Description of how study procedures (e.g. intervention or diagnosis) compare to standard of care, including description of alternative treatments, procedures, or methods of diagnosis**
    There will be no study procedures.

  - **Description of what happens to participants receiving therapy when study ends or if a participant's participation in the study ends prematurely.**

Not applicable.

- **For studies that are conducted entirely internationally, describe the nature of the involvement of investigators at each site in the study, i.e., indicate which personnel will be present at the international site(s) and describe the nature of their involvement, including which staff will have direct contact with subjects and/or perform any study related procedures and which staff will have access to study data and perform data analysis**
  There will be no transfer of data to an outside institution for this study.

- **Remuneration as applicable. Indicate if payments to subjects are made upon completion of study visits/certain procedures and how remuneration is pro-rated, particularly for non-completers**
  We will provide renumeration of $48 (gift card purchased through MGB approved mechanisms or check, depending on participant preference).

- **Description of plans for sending and/or receiving specimens or data with research collaborators outside Mass General Brigham or with NIH (e.g., dbGaP) or other tissue/data repositories (include details of identified versus de-identified sharing, how data or specimens are labeled/coded, secure transfer method, external IRB approval as applicable, storage for future use, secure transfer method, etc.)**
  No transfer of data to an outside institution will be for this study.

7. Risks and Discomforts

Provide detailed description of potential risks of each study-related procedure/intervention, including:

- **Complications of surgical and non-surgical procedures**
  Not applicable.

- **Drug side effects and toxicities**
  Not applicable.

- **Device complications/malfunctions**
  Not applicable.

- **Psychosocial risks**
  The participants will be introduced or re-initiated on the topic of ACP. Some patients with serious illness do not wish to discuss ACP because they feel uncomfortable with the topic. This potential discomfort, however, is a part of the routine practice in the ED. Many ED clinicians discuss ACP with patients routinely, and the study does not add additional discomfort that is different from the routine clinical practice. Further, the PI is trained in serious illness communication and will be available to manage patients' anxiety and other emotions as needed.

- **Privacy/confidentiality risks**
  Loss of confidentiality is a minor risk. The protection of data for this study includes the following:

  1) Study data will be collected and managed using Dropbox Business, which is fully encrypted and is compliant with Partners' policies and procedures. Data pertinent to this study will only be given access to only the members of our study team approved by the IRB.

2) REDCap electronic data capture tools, which are hosted at BWH[1]. REDCap (Research Electronic Data Capture) is a secure, web-based application designed to support data capture for research studies, thereby providing an intuitive interface for validated data entry, audit trails for tracking data manipulation and export procedures, automated export procedures for seamless data downloads to common statistical packages, and procedures for importing data from external sources.

3) Study participants will be identified by a unique numeric identification, and their protected health information (PHI) will be de-identified.

4) Study participant PHI will be collected and stored on a secure REDCap survey, which will be created on a secure, password protected desktop computer located at BWH where only the study staff will have access.

5) At study completion, contact information and the link between the identifying information and their data will be destroyed.

6) Finally, to ensure that all staff is compliant with confidentiality training, all study staff has completed the CITI course for the protection of human subjects training in research ethics and good clinical practice.

- **Genetic research risks**
  Not applicable.

- **Radiation risks**
  Not applicable.

- **Include description of steps taken to decrease relevant risks, including the following:**
  - **How procedures used are consistent with sound research design and do not expose unnecessary risks**
    Verbal consent will be obtained from the patient / caregiver participants. After learning about the general background of the study and reading the study information sheet (electronic or hardcopy), the potential participants will be able to decline to participate. Further, if the participant expressed anxiety or distress from the intervention (determined by the treating clinician), he/she is offered to discontinue the study and the PI will be responsible to care for the participant's emotional needs. The PI will also contact the patient's primary outpatient clinician to ensure adequate follow up of the patient after leaving the ED.

  - **When appropriate, researchers use procedures already being performed on subjects for diagnostic or treatment purposes**
    Not applicable.

8. Benefits

Provide detailed description of potential benefits of study participation, including:

- **Either describe potential benefits to participating individuals or clearly state that there is no direct benefit to individuals**

No definitive benefit has been demonstrated. However, our prior pre/post-intervention studies indicated that the intervention may lead to increased ACP engagement in patients with serious illness (see **Previous Studies** section above).

- **If multiple subject populations are to be enrolled, describe any differences between groups with regard to potential benefit (e.g. potential for benefit to an affected subject population versus no potential benefit for healthy controls, etc.)**

Based on the demographic data that we have thusfar, we do not anticipate differences between groups with regard to the potential benefit.

- **Potential benefits to society (e.g., increased understanding of disease process, etc.)**

For our patients, we hope that our intervention will lead patients to have more ACP conversations with their outpatient clinicians and loved ones. ACP conversations can lead to well-informed shared decision making and improved quality of life at the end of life.[1] For the society, ACP conversations are associated with lower rates of in-hospital death, less aggressive medical care at the end of life, earlier hospice referrals, increased peacefulness, and a 56% greater likelihood to have end-of-life wishes known and followed.[1-8] Furthermore, patients with documented ACP conversations experience a 36% reduction in the cost of end-of-life care, with an average cost savings of $1,041 per patient in the last week of life.[9]

9. Statistical Analysis

Describe plans for statistical analysis, including:

- **Statistical methods/data analysis plan**

Aim 1 quantitative data analysis:

We will conduct an intention-to-treat analysis. Since the primary outcome is mean change in self-reported ACP engagement, we do not expect differences among patients who are admitted vs. discharged. For our secondary outcome, we suspect that there is a propensity of more ACP documentation in patients who are hospitalized and missingness in patients who are primarily cared for at outside institutions. We will plan to use regression in subgroup analysis for ACP documentation to account for hospitalization status. In addition, we will perform sensitivity analyses to account for clustering by individual outpatient clinicians, palliative care consultations, and cognitive impairment status. We will also perform sensitivity analyses for survey respondents (e.g., patients vs. caregivers). We will compare patient demographics by study arm to assess randomization using bivariable analyses (t-test for continuous variables and chi-square for categorical variables). The mean changes in ACP engagement (primary outcome) at one month will be compared by study arms to estimate effect sizes. Within arms, we will use a one-sample binomial exact test of proportions for categorical outcomes (e.g., EMR documentation), and Wilcoxon signed ranks test for ordinal outcomes (e.g., ACP Engagement Survey) at baseline, after the intervention at one month. A p-value of 0.05 will be the significance threshold. To address missing data, I will conduct a secondary analysis using linear mixed models at baseline and one month. For qualitative outcomes,

Aim 2 qualitative data analysis:

We will conduct in-depth, semi-structured interviews with participants. We will explore the perceived benefits of the intervention as well as barriers to completing ACP conversations after leaving the ED. The interviews will be recorded and professionally transcribed. The transcripts will be analyzed to identify recurring themes that illustrate the qualiative benefits (e.g., how the intervention changed their lives) and barriers to completing ACP conversations (e.g., how attempted ACP conversations were unsuccessful). Transcripts will be analyzed by two trained researchers to ensure interpretive integrity and consistency per standard comprehensive qualitative analysis methods. We will use

framework analysis as the analytic approach,[33] which allows the flexibility of incorporating *a priori* considerations as well as more emergent themes in the data. The coding structure will be collaboratively and iteratively developed by the research team and include both prefigured and emergent codes.[34] We will use an ethnographic data management software program, NVivo 10 (QSR International). If there are disagreements, the mentors/advisors and we will review the quotes and themes to discuss their meaning and to achieve consensus. We will follow the consolidated criteria for reporting qualitative research.[35]

- **Power analysis (e.g., sample size, evaluable subjects, etc.)**

For Aim 1 (quantitative data):
We will compare 1-, 3-, 6-month ACP outcomes in the intervention arm to the control arm to estimate the preliminary effect sizes. Our prior study demonstrated a mean patient-reported ACP engagement increased from 3.8 to 4.3 on a 5-point scale, corresponding to a moderate effect size of 0.50.[36] A conservative estimate of effect size is 0.25. we also expect that 10% of enrolled patients will die before completing all follow-ups. With a sample size of 60 patients per group, we would have 90% power to detect the difference using a 2-sided Fisher's exact test (alpha=0.05). We plan to exclude patients who die before 6-month in the analysis.

For Aim 2 (qualitative data):
Based on my previously successful study, in which I explored patients' attitudes about *ED GOAL*,[37] interviewing ~10 dyads of patients and caregivers (N~20 total) with a diverse representation of perspectives will yield thematic saturation for tailoring *ED GOAL*. However, I will continue to enroll more participants if thematic saturation is not achieved.

10. Monitoring and Quality Assurance

Describe the plans that will be followed by study staff for monitoring and quality assurance, including:

- **Adverse event criteria and reporting procedures**
  Any AE will be reported to our institutional review board at the time of annual continuing review by the principal investigator using an Adverse Event Form in accordance with the Partners Human Research Committee guidelines for AE reporting.

- **Planned safety monitoring, e.g., data monitoring committee (DMC)/data and safety monitoring board (DSMB), independent monitor, PI-monitored, etc., including planned frequency of review. If DMC/DSMB monitored, include either charter as separate attachment or complete DMC/DSMB APPENDIX**
  The proposed research is a minimal risk study and does not meet the criteria for an NIH-defined Phase III trial given no patient outcomes will be collected. Therefore, a data and safety monitoring board will not be required, and PI will be solely responsible for data and safety monitoring of the proposed research. In the event of an adverse event, the research team will report immediately to the PI, who will be responsible for management and reporting to the IRB, and to the NIH institute.

- **Outcomes monitoring, including planned frequency of review.**
  Not applicable.

- **Study stopping rules as applicable**
  Unanticipated Problems (UP): As with any AE above, the PI will report any UP within 5 working days / 7 calendar days of the date. The investigator first becomes aware of the problem using an

Adverse Event Form in accordance with the Partners Human Research Committee guidelines for AE reporting Please see attached institutional policy.

- **Internal monitoring of source data, protocol adherence, and recordkeeping, including which staff will be responsible and planned frequency of review**
  The PI is responsible for monitoring and recordkeeping on REDCap and qualitative interview findings.

- **Independent monitoring of source data as applicable**
  All personal health information will be removed from the research data from the participants and clinicians at the earliest possible time. All data will be stored in a secured Partners approved shared drive accessible only to the study staff. Recordings will be stored on an encrypted recording device and saved to the secure Partners-approved shared drive.

11. Privacy and Confidentiality

- **Select the Privacy and Confidentiality measures that apply to this research by checking the box next to each statement (Check all that apply)**
- **Note that not all of the measures outlined below may apply to your study**
- **Do not delete statements that do not apply to your study; leave the boxes unchecked**
- **Describe any additional privacy and/or confidentiality measures that are not captured by the check box items in free text following the check boxes**

☒ Study procedures will be conducted in a private setting
☒ Only data and/or specimens necessary for the conduct of the study will be collected
☒ Data collected (paper and/or electronic) will be maintained in a secure location with appropriate protections such as password protection, encryption, physical security measures (locked files/areas)
☐ Specimens collected will be maintained in a secure location with appropriate protections (e.g. locked storage spaces, laboratory areas)
☒ Data and specimens will only be shared with individuals who are members of the IRB-approved research team or approved for sharing as described in this IRB protocol
☒ Data and/or specimens requiring transportation from one location or electronic space to another will be transported only in a secure manner (e.g. encrypted files, password protection, using chain-of-custody procedures, etc.)
☒ All electronic communication with participants will comply with Mass General Brigham secure communication policies
☒ Identifiers will be coded or removed as soon as feasible and access to files linking identifiers with coded data or specimens will be limited to the minimal necessary members of the research team required to conduct the research
☒ All staff are trained on and will follow the Mass General Brigham policies and procedures for maintaining appropriate confidentiality of research data and specimens
☒ The PI will ensure that all staff implement and follow any Research Information Service Office (RISO) requirements for this research
☒ Additional privacy and/or confidentiality protections

12. References

1. Wright AA, Zhang B, Ray A, et al. Associations between end-of-life discussions, patient mental health, medical care near death, and caregiver bereavement adjustment. *JAMA.* 2008;300(14):1665-1673.
2. Ray A, Block SD, Friedlander RJ, Zhang B, Maciejewski PK, Prigerson HG. Peaceful awareness in patients with advanced cancer. *J Palliat Med.* 2006;9(6):1359-1368.
3. Detering KM, Hancock AD, Reade MC, Silvester W. The impact of advance care planning on end of life care in elderly patients: randomised controlled trial. *BMJ.* 2010;340:c1345.
4. Khandelwal N, Kross EK, Engelberg RA, Coe NB, Long AC, Curtis JR. Estimating the effect of palliative care interventions and advance care planning on ICU utilization: a systematic review. *Crit Care Med.* 2015;43(5):1102-1111.
5. Lakin JR, Block SD, Billings JA, et al. Improving Communication About Serious Illness in Primary Care: A Review. *JAMA Intern Med.* 2016;176(9):1380-1387.
6. Dixon J, Matosevic T, Knapp M. The economic evidence for advance care planning: Systematic review of evidence. *Palliat Med.* 2015;29(10):869-884.
7. Khandelwal N, Benkeser DC, Coe NB, Curtis JR. Potential Influence of Advance Care Planning and Palliative Care Consultation on ICU Costs for Patients With Chronic and Serious Illness. *Crit Care Med.* 2016;44(8):1474-1481.
8. Shen MJ, Prigerson HG, Paulk E, et al. Impact of end-of-life discussions on the reduction of Latino/non-Latino disparities in do-not-resuscitate order completion. *Cancer.* 2016;122(11):1749-1756.
9. Zhang B, Wright AA, Huskamp HA, et al. Health care costs in the last week of life: associations with end-of-life conversations. *Arch Intern Med.* 2009;169(5):480-488.
10. Smith TJ, Coyne P, Cassel B, Penberthy L, Hopson A, Hager MA. A high-volume specialist palliative care unit and team may reduce in-hospital end-of-life care costs. *J Palliat Med.* 2003;6(5):699-705.
11. Mack JW, Cronin A, Keating NL, et al. Associations between end-of-life discussion characteristics and care received near death: a prospective cohort study. *J Clin Oncol.* 2012;30(35):4387-4395.
12. Smith AK, McCarthy E, Weber E, et al. Half of older Americans seen in emergency department in last month of life; most admitted to hospital, and many die there. *Health Aff (Millwood).* 2012;31(6):1277-1285.
13. Wilber ST, Blanda M, Gerson LW, Allen KR. Short-term functional decline and service use in older emergency department patients with blunt injuries. *Acad Emerg Med.* 2010;17(7):679-686.
14. Deschodt M, Devriendt E, Sabbe M, et al. Characteristics of older adults admitted to the emergency department (ED) and their risk factors for ED readmission based on comprehensive geriatric assessment: a prospective cohort study. *BMC Geriatr.* 2015;15:54.
15. Nagurney JM, Fleischman W, Han L, Leo-Summers L, Allore HG, Gill TM. Emergency Department Visits Without Hospitalization Are Associated With Functional Decline in Older Persons. *Ann Emerg Med.* 2017;69(4):426-433.
16. Steinhauser KE, Christakis NA, Clipp EC, McNeilly M, McIntyre L, Tulsky JA. Factors considered important at the end of life by patients, family, physicians, and other care providers. *JAMA.* 2000;284(19):2476-2482.
17. Oulton J, Rhodes SM, Howe C, Fain MJ, Mohler MJ. Advance directives for older adults in the emergency department: a systematic review. *J Palliat Med.* 2015;18(6):500-505.
18. O'Connor AE, Winch S, Lukin W, Parker M. Emergency medicine and futile care: Taking the road less travelled. *Emerg Med Australas.* 2011;23(5):640-643.

19. Stone SC, Mohanty S, Grudzen CR, et al. Emergency medicine physicians' perspectives of providing palliative care in an emergency department. *J Palliat Med.* 2011;14(12):1333-1338.
20. Smith AK, Fisher J, Schonberg MA, et al. Am I doing the right thing? Provider perspectives on improving palliative care in the emergency department. *Ann Emerg Med.* 2009;54(1):86-93, 93 e81.
21. Ouchi K, George N, Revette AC, et al. Empower seriously ill older adults to formulate their goals for medical care in the emergency department. *J Palliat Med.* 2018;In Press.
22. Leiter R, Yusufov, M, Hasdianda, MA, Fellion, L, Reust A, Tulsky, JA, Block, SD, Ouchi, K. . Fidelity and feasibility of a brief emergency department intervention to empower adults with serious illness to initiate advance care planning conversations. *J Pain Symptom Manage.* 2018;In Press.
23. Ouchi K, Strout T, Haydar S, et al. Association of Emergency Clinicians' Assessment of Mortality Risk With Actual 1-Month Mortality Among Older Adults Admitted to the Hospital. *JAMA Netw Open.* 2019;2(9):e1911139.
24. Ouchi K, Jambaulikar G, George NR, et al. The "Surprise Question" Asked of Emergency Physicians May Predict 12-Month Mortality among Older Emergency Department Patients. *J Palliat Med.* 2018;21(2):236-240.
25. Marcantonio ER, Ngo LH, O'Connor M, et al. 3D-CAM: derivation and validation of a 3-minute diagnostic interview for CAM-defined delirium: a cross-sectional diagnostic test study. *Ann Intern Med.* 2014;161(8):554-561.
26. Galvin JE. The Quick Dementia Rating System (Qdrs): A Rapid Dementia Staging Tool. *Alzheimers Dement (Amst).* 2015;1(2):249-259.
27. Borson S, Scanlan JM, Chen P, Ganguli M. The Mini-Cog as a screen for dementia: validation in a population-based sample. *J Am Geriatr Soc.* 2003;51(10):1451-1454.
28. Borrelli B. The assessment, monitoring, and enhancement of treatment fidelity in public health clinical trials. *J Public Health Dent.* 2011;71 Suppl 1:S52-63.
29. Sudore RL, Stewart AL, Knight SJ, et al. Development and validation of a questionnaire to detect behavior change in multiple advance care planning behaviors. *PLoS One.* 2013;8(9):e72465.
30. Van Scoy LJ, Day AG, Howard M, Sudore R, Heyland DK. Adaptation and Preliminary Validation of the Advance Care Planning Engagement Survey for Surrogate Decision Makers. *J Pain Symptom Manage.* 2019;57(5):980-988 e989.
31. Gramling R, Stanek S, Ladwig S, et al. Feeling Heard and Understood: A Patient-Reported Quality Measure for the Inpatient Palliative Care Setting. *J Pain Symptom Manage.* 2016;51(2):150-154.
32. Engelberg R, Downey L, Curtis JR. Psychometric characteristics of a quality of communication questionnaire assessing communication about end-of-life care. *J Palliat Med.* 2006;9(5):1086-1098.
33. Pope C, Ziebland S, Mays N. Qualitative research in health care. Analysing qualitative data. *BMJ.* 2000;320(7227):114-116.
34. J.W. C. *Qualitative Inquiry & Research Design: Choosing among Five Approached.* 3rd edition ed: Sage Publications; 2013.
35. Tong A, Sainsbury P, Craig J. Consolidated criteria for reporting qualitative research (COREQ): a 32-item checklist for interviews and focus groups. *Int J Qual Health Care.* 2007;19(6):349-357.
36. Shi Y, Barnes DE, Boscardin J, et al. Brief English and Spanish Survey Detects Change in Response to Advance Care Planning Interventions. *J Pain Symptom Manage.* 2019.
37. Ouchi K, George N, Revette AC, et al. Empower Seriously Ill Older Adults to Formulate Their Goals for Medical Care in the Emergency Department. *J Palliat Med.* 2019;22(3):267-273.

APPENDIX A

Data Monitoring Committee / Data and Safety Monitoring Board Appendix

---

- *To be completed for studies monitored by Data Monitoring Committee (DMC) or Data and Safety Monitoring Board (DSMB) if a full DMC/DSMB charter is not available at the time of initial IRB review.*
- *DMC/DSMB Charter and/or Roster can be submitted to the IRB later via Amendment, though these are not required.*

A Data Monitoring Committee (DMC) or Data and Safety Monitoring Board (DSMB) will be convened for safety monitoring of this research study. The following characteristics describe the DMC/DSMB convened for this study (Check all that apply):

☐ The DMC/DSMB is independent from the study team and study sponsor.

☐ A process has been implemented to ensure absence of conflicts of interest by DMC/DSMB members.

☐ The DMC/DSMB has the authority to intervene on study progress in the event of safety concerns, e.g., to suspend or terminate a study if new safety concerns have been identified or need to be investigated.

☐ Describe number and types of (i.e., qualifications of) members:
Click or tap here to enter text.

☐ Describe planned frequency of meetings:
Click or tap here to enter text.

☐ DMC/DSMB reports with no findings (i.e., "continue without modifications") will be submitted to the IRB at the time of Continuing Review.

☐ DMC/DSMB reports with findings/modifications required will be submitted promptly (within 5 business days/7 calendar days of becoming aware) to the IRB as an Other Event.
