# Supplementary material for: Serious Illness Conversations in the Emergency Department for Older Adults With Advanced Illnesses: A Randomized Clinical Trial
Source: JAMA Netw Open. 2025 Jun 18;8(6):e2516582. doi: 10.1001/jamanetworkopen.2025.16582 (PMC12177648; doi:10.1001/jamanetworkopen.2025.16582)
Supplement: Supplement 2. — eAppendix. ED GOAL Intervention Script [file jamanetwopen-e2516582-s002.pdf]

Supplementary Online Content

Ouchi K, Block SD, Rentz DM, et al. Serious illness conversations in the emergency department for older adults with advanced illnesses: a randomized clinical trial. *JAMA Netw Open.* 2025;8(6):e2516582. doi:10.1001/jamanetworkopen.2025.16582

eAppendix. ED GOAL Intervention Script

This supplementary material has been provided by the authors to give readers additional information about their work.

© 2025 Ouchi K et al. *JAMA Network Open*.

eAppendix. ED GOAL Intervention Script

ED GOAL^{CG}

I. For patients with <u>mild cognitive impairment (MCI)</u> and <u>mild dementia</u> and their caregivers

Before *ED GOAL*:

<u>1. Ask for preference regarding involving the caregiver</u>

To the patient:

"Since I will be asking you questions about advance care planning, would you prefer that I involve your (caregiver)?"

<u>2. Acknowledge participation from the caregiver</u>

To the caregiver:

"Thank you for joining us – we recognize that you are a big part of the patient's life. Are you ok with talking to us both?"

** Look for non-verbal answers/communication from the caregivers to decern if the patient or caregivers may be afraid/concerned about talking in front of each other.**

e.g., #1 "<u>In this moment</u>, can you share your perspective about what I asked?"

e.g., #2 "<u>If not now</u>, may be later by whatever means easiest for you?"

e.g., #3 "<u>If not now</u>, may be you can share when/how for us to communicate about your perspective about what I asked?"

<u>3. Emphasize the potential need to validate or prompt for response from the patient</u>

To the patient and caregiver:

"We may ask (caregiver) for his/her perspective as we proceed with our interview. Is that ok?"

During *ED GOAL*

<u>1. When questions are not appropriately answered</u>

a. Ask the patient:

"I want to understand more about what you are saying – could you explain this a little more to me? (can the patient take it one step further? Give patient one more chance to express before going to the caregiver)"

b. Ask for permission from the patient

- "would it be ok if I checked in with (caregiver)?"

c. Ask the caregiver

– "What is your perspective on what (patient) just said? Is that your understanding of what I asked?"

© 2025 Ouchi K et al. *JAMA Network Open*.

e.g., #1 "In this moment, can you share your perspective about what I asked?"

© 2025 Ouchi K et al. *JAMA Network Open*.

e.g., #2 "If not now, may be later by whatever means easiest for you?"

e.g., #3 "If not now, may be you can share when/how for us to communicate about your perspective about what I asked?"

If the interventionist thinks that the patient is answering questions appropriately, still give the chance for the caregivers to weigh in with b and c above.

After *ED GOAL*:

1. Partner with caregiver

To the caregiver:

"For the best results, it is important that both you and I work together with the (patient). What questions do you have about what we just discussed?"

2. Re-emphasize with the caregiver that we will be checking in:

To the caregiver:

"In addition to providing information to (patient), we would also like you to review the helpful information sheet with you."

3. Outline for the next steps

"As a guide, we would like you to use this sheet to talk to each other."

Patient's job: "While it may be hard, serious illness questions help patients think through this topic. It could be beneficial to you to answer these questions. Would you consider them?"

Caregiver's job: "You should try to help (patient) answer these questions."

Study clinician's job: "I will help you document this on this sheet and communicate to your doctor."

© 2025 Ouchi K et al. *JAMA Network Open*.

II. For patients with moderate and severe dementia and their caregivers

Before *ED GOAL*:

1. Address the patient first

To the patient (when appropriate):

“Thank you for joining and allowing us to talk to (caregiver), and we will ask you for your input as well!”

2. Ask the caregiver about the patient presence

To the caregiver (when appropriate):

“Would you think it would be appropriate to include (patient) in our conversation today?”

If “Yes” → include the patient

If “No” → do not include the patient, say “thank you” to the patient and ask the caregiver to escort the patient out.

3. Emphasize the potential need to validate or prompt for response from the patient

To the patient and caregiver:

“We will ask for everyone’s perspective as we proceed with our interview. Is that ok?”

**** *ED GOAL* as usual with the caregivers who are the subjects of our research. ****

© 2025 Ouchi K et al. *JAMA Network Open*.

Brief Motivational Interview ED Intervention to Facilitate Serious Illness Communication

1) <u>Open:</u>

In the ED, we care for patients in the moment and **also help them prepare** for what's ahead after leaving the ED.

First of all, there is **no new information** regarding your (*serious illness*).

Because you have (*serious illness*) and you are in the ED, I am **worried** that things could get worse at some point in the future. *EXPECT EMOTION*

What is your **understanding** now of where you are with your illness?

Connect emotionally upfront:

Option 1 – What thoughts have you had about getting sicker in the future?

Option 2 – What worries have you had about your (*serious illness*)?

This is a good time to begin thinking about what healthcare is right for you **if you get sicker**.

Would it be okay if I help you think through how to talk to Dr. (*name*) about what you want in your care in the future?

<u>2) Build Rapport (may skip if already discussed above):</u>
Option 1 - Tell me about your (*serious illness*) and how it affects your life?
Option 2 – What worries have you had about your (*serious illness*)?

<u>3) Information & Feedback</u>

Elicit (Ask): Tell me how much Dr. (*name*) understands about how (*serious illness*) is affecting you?

Provide (Tell): Telling your doctor what is important to you will help you get the care you want in the future.

Elicit (Ask): What do you think?

<u>4) Assess Readiness</u>

How ready are you to share what is important to you with Dr. (*name*)?

Not Ready ---------------- *Somewhat Ready* ----------------- *Completely Ready*

Reinforce positives: Sounds like you are (*Not / Somewhat / Completely*) ready to talk about this with Dr. (*name*). Thank you for sharing that.

Ask about reason: What *would* make you be even more ready?

<u>5) Reflect & Summarize</u>

What I heard you say is that:

Your (*serious illness*) affects your life in _____ ways.

You think that exploring what is important to you is ____.

You are (*Not / Somewhat / Completely*) ready to start exploring the right type of care with Dr. (*name*) **because** ______.

Did I get that correctly?

<u>6) Action Plan</u>
Ask next steps: What do you think might be helpful for you to do before you see Dr. (*name*)?

Ask permission: That sounds great. **Can I add something?**

Make a recommendation: What I heard you say is that:

<u>a. NOT ready</u> "This isn't the right time for you to talk to your doctor about your future care. Here is an information sheet that you may find helpful to read at home before your next visit with your doctor. This might help you think if this could be helpful to you."

© 2025 Ouchi K et al. *JAMA Network Open*.

b. Somewhat or completely ready “You are somewhat ready to explore the right type of care for you with Dr. (*name*). I would like to review questions that many patients find useful to think about before your next doctor’s visit.”
**Ask Serious Illness Questions**
**Closing:** I will also **notify your doctor** to let him/her know about your visit to the ED and our conversation today. **Is that okay?** Thank you for talking with me.

© 2025 Ouchi K et al. *JAMA Network Open*.

Serious illness questions for *ED GOAL* clinicians

*Not evidence-based questions. Evaluate if clinically appropriate to use.

Components	What to ask
**What's important** should health worsen	"What's important to you if your health worsens?"
**Hopes** for the future	"What hopes do you have about your illness?"
**Worries** about the future	"What worries do you have about getting sicker?"
**Trade-offs**	"How much more would you be willing to go through for the possibility of more time?"
**Minimal quality of life** considered acceptable to live	"What is the minimum quality of life you would consider living?"
Health states **worse than dying***	"What health states would you consider worse than dying itself?"
**Intubation** preferences* (Do not ask unless patient brings it up)	"What thoughts do you have about being kept alive on machines?"
**CPR** preferences* (Do not ask unless patient brings it up)	"What thoughts do you have about naturally dying?"
**Location** at the end of life*	"What thoughts do you have about where you want to be if you were dying?"
**Awareness** of loved ones	"How much do your loved ones know about what you shared with me?"

© 2025 Ouchi K et al. *JAMA Network Open*.

Intervention Fidelity Checklist

Study ID ________________________________

Received interview?
- ○ No (control)
- ○ Yes (intervention)

BMI Script

[Attachment: "BMI script.pdf"]

Enrolling clinician: [enrolling_clinician]

Assessor #1: [enrolling_ra]

Assessor #2: ________________________________

1. Opening

Setting up the topic (not related to ED care)
- Y
- N

Communicate importance of future care planning
- ○ Y
- ○ N

Ask permission to discuss
- ○ Y
- ○ N

____

2. Rapport building

Ask open-ended question
- ○ Y
- ○ N

3. Information & Feedback

Elicit current thoughts about communicating goals of care
- ○ Y
- ○ N

Provide facts
- ○ Y
- ○ N

Sum up and restate in patient's own words
- ○ Y
- ○ N

____

© 2025 Ouchi K et al. *JAMA Network Open*.

**4. Readiness**	
Assess readiness	◯ Y<br>◯ N
Reinforce positives	◯ Y<br>◯ N
Ask what would make it more ready?	◯ Y<br>◯ N

____

**5. Summary**	
Summarize patient's responses	◯ Y<br>◯ N
Confirm patient's responses	◯ Y<br>◯ N

____

**6. Action**	
Elicit specific next step from patient's perspective	◯ Y<br>◯ N
Make a recommendation	◯ Y<br>◯ N
Ask permission to communicate to the outpatient clinician	◯ Y<br>◯ N

____
(Total score of < 70/100 needs retraining)

**Part II Communication Skills**	
Language appropriate	◯ 0<br>◯ 1<br>◯ 2<br>◯ 3<br>◯ 4<br>◯ 5

© 2025 Ouchi K et al. *JAMA Network Open*.

Reflective listening	◯ 0<br>◯ 1<br>◯ 2<br>◯ 3<br>◯ 4<br>◯ 5
Respect	◯ 0<br>◯ 1<br>◯ 2<br>◯ 3<br>◯ 4<br>◯ 5
Negotiation	◯ 0<br>◯ 1<br>◯ 2<br>◯ 3<br>◯ 4<br>◯ 5
Respond to emotions	◯ 0<br>◯ 1<br>◯ 2<br>◯ 3<br>◯ 4<br>◯ 5
Assessing mutual understanding	◯ 0<br>◯ 1<br>◯ 2<br>◯ 3<br>◯ 4<br>◯ 5
Listening for cues	◯ 0<br>◯ 1<br>◯ 2<br>◯ 3<br>◯ 4<br>◯ 5
Redirects when needed	◯ 0<br>◯ 1<br>◯ 2<br>◯ 3<br>◯ 4<br>◯ 5

© 2025 Ouchi K et al. *JAMA Network Open*.

***ED GOAL* Data Abstraction Manual:**

Patient Status:

1. Patient status at 1 month and 6 months after intervention (alive/deceased)
    a. If deceased, record "Date of Death"
        i. Date is listed if you hover over their name on the upper left side of patient's chart

Encounter Details:

1. Fill out "ED Disposition" only
    a. Chart Review-> Encounters-> Filters by "Emergency Medicine"->Open relevant ED visit provider notes->Find Disposition on the right

Change in MOLST Form Documentation:

Hover over yellow code banner and look for MOLST under "Advance Care Planning Documents"

1. MOLST on file prior to intervention? (Yes/No)
2. MOLST on file prior to discharge, during this admission (if intervention occurred before hospitalization)? (Yes/no)
    a. This should be left blank if the patient did not enroll in person
3. MOLST on file @1 month, 3 month, 6 months, and 12 months post intervention? (yes/no)
4. Date of first MOLST documentation?
    a. This is the date the MOLST was signed (not date it was entered into EMR). Click and open MOLST form to see signed date.
5. Date of final MOLST documentation?
    a. If MOLST has been uploaded more than once, click the file to see if there are any changes. If forms are different document both.

Change in Serious Illness Conversation Documentation:

1. Serious illness conversation on file @1 month, 3 month, 6 months, and 12 months post intervention?
    a. *Search MRN on LabArchives -> SIC Abstractions Page -> corresponding f/u time point. All conversation dates and "context" are documented here.*
    b. Record date if there is a change. Add context by clicking the small chat bubble to the left of the date. Redact all PHI from the note.
2. Date of Final SIC documentation
    a. If patient had a serious illness conversation during the intervention, enter the most recent date here.

Change in HCP Documentation:

1. HCP documented in EMR prior to intervention? (Yes/No)
2. New HCP documentation prior to discharge, during admission (if intervention occurred before hospitalization)? (Yes/No)
    a. This should be left blank if the patient did not enroll in person
3. Change in HCP documentation @1 month, 3 month, 6 months, and 12 months post intervention?
    b. *Hover over yellow code banner on upper left side and check for Healthcare Proxy under "Advance Care Planning Documents"*
    c. Record date if there is a change

© 2025 Ouchi K et al. *JAMA Network Open*.

    d. HCP documents can be listed as “Healthcare Proxy” OR “Advanced Directives.” Click into each form to see if it is an HCP form and if there is a new documentation uploaded or if an old one was just re-uploaded.
4. Final date of HCP documentation?
    e. If patient has HCP documentation during intervention, enter the most recent date here.

Deceased Patients:

1. Keep a cumulative count of post-enrollment healthcare utilization, even if they were deceased at a certain timepoint.
2. Yes/no questions should be answered as “N/A - Patient deceased”
3. For patient code status, choose “N/A - Patient deceased” for time points in which they are not alive
4. For Change in MOLST form, Changes in Serious Illness Conversation Status, and Change in Healthcare Proxy (HCP) Documentation sections, choose “N/A, Patient deceased” for timepoints where patients are not alive.

Patients that Enrolled in Person:

1. The following will only be filled out for patients that were enrolled in person and admitted to the hospital through the ED:
    a. Code Status Changes During Hospital Stay (if intervention occurs before hospitalization)
    b. MOLST on file prior to discharge, during this admission (if intervention occurred before hospitalization)? (Yes/no)
    c. New HCP documentation prior to discharge, during admission (if intervention occurred before hospitalization)? (Yes/No)

© 2025 Ouchi K et al. *JAMA Network Open*.

Serious Illness Conversation Documentation Codebook

Blinded RA: Search for each term, individually or in small groups using "or" between phrases:

a. Prognosis / prognostic information
b. Goals of care / GOC
c. ACP/Advance Care Planning
d. Code Status
e. Hopes
f. Worries / Fears
g. Quality of Life / QOL
h. Palliative
i. Serious illness conversation / SIC
j. Discussion
k. Understanding of illness / patient illness understanding
l. Tradeoffs
m. Worse than dying
n. Resuscitation
o. Intubations
p. Wishes
q. Preferences
r. MOLST
s. DNR/DNI
t. Hospice
u. Spirituality / God / Faith
v. Comfort measures / CMO
w. Critical abilities / abilities critical to your life
x. "What's important"
y. "More time"
z. "Be at home"
aa. Trust/support
bb. Pain management

Cut/Paste into Search:

Prognosis **or** prognostic information **or** Goals of care **or** ACP **or** Advance Care Planning **or** Code Status **or** Hopes **or** Worries **or** Fears **or** Quality of Life or QOL **or** Palliative **or** Serious illness conversation **or** SIC **or** Discussion **or** Understanding of illness **or** patient illness understanding or Tradeoffs **or** Resuscitation **or** Intubation **or** Wishes or Preferences **or** MOLST or DNR/DNI **or** Hospice or Spirituality **or** God or Faith **or** GOC **or** Comfort measures **or** CMO **or** Critical abilities **or** abilities critical to your life **or** What's important **or** More time **or** Be at home or Trust or support **or** Pain management

Inclusion criteria:

1. Serious illness conversation and/or understanding, advance care planning, goals/values pertaining *specifically* to priorities for treatment or outcomes of treatment.
2. Limitations to care including, code status, resuscitation, defibrillation, intubation, escalation of care, renal replacement therapy, artificial nutrition, or hydration.
3. Hospice, comfort measures, stopping disease-modifying therapies, or de-escalating of care.

Examples of Inclusions:

- *"I had an honest conversation with the patient about their prognosis and their goals of care, we discussed how it was most important to him to be at home and with this family if time were short."*

© 2025 Ouchi K et al. *JAMA Network Open*.

- *“We discussed what matters most to the patient, wants to be at home and with family”*
- *“Together we reviewed the MOSLT form, I recommended against intubation given her underlying lung disease."*
- *“Given how severe her side effects were after the last round of chemo therapy we discussed the option of stopping further cancer directed therapy and focusing on ensuring her comfort and quality of life with is her priority at this time.”*

<u>Exclusion criteria:</u>

Note documented by the *ED GOAL* study interventionists do not count unless they are copied and pasted into a new note with a different author AND the new note states that ACP was reviewed/discussed.

The simple mention of code status (“Full Code”), note title (“Advance Care Planning Note”), or automatic phrases populated by the medical records do not count as positive for SIC.

<u>Examples of Exclusion:</u>

- *“Code Status: Full Code.”*
- *“MOLST on file.”*
- *“Patient could benefit from code status discussions.”*
- *“Family states living will is completed.”*

Scan/read notes to determine if there is a new SIC. If yes, document the date, phrases found in the note, and author of the note in the appropriate date range in the REDCap in this file.

<u>If uncertain about findings:</u>

If the blind RA is not sure about the presence/absence of SIC, the notes/phrases in questions are reviewed by other blinded study team members to achieve consensus.

<u>Training new RA:</u>

When training a new blinded RA to do SIC chart abstraction, select 5-8 patients for both RAs to review with approximately 20-30 total time points. Train/retrain until interrater reliability reaches 90% or higher concordance/agreement.

© 2025 Ouchi K et al. *JAMA Network Open*.
