# Supplementary material for: Serious Illness Conversations in the Emergency Department for Older Adults With Advanced Illnesses: A Randomized Clinical Trial
Source: JAMA Netw Open. 2025 Jun 18;8(6):e2516582. doi: 10.1001/jamanetworkopen.2025.16582 (PMC12177648; doi:10.1001/jamanetworkopen.2025.16582)
Supplement: Supplement 3. — Data Sharing Statement [file jamanetwopen-e2516582-s003.pdf]

Data Sharing Statement

Ouchi. Serious Illness Conversations in the Emergency Department for Older Adults With Advanced Illnesses. *JAMA Netw Open*. Published June 18, 2025.
doi:10.1001/jamanetworkopen.2025.16582

Data
Additional Information: clinicaltrials.gov, https://clinicaltrials.gov/, NCT05209880
Data available: Yes
Data types: Deidentified participant data
How to access data: Please contact the principal investigator for the data request.
When available: With publication

Supporting Documents
Document types: Statistical/analytic code, Informed consent form
How to access documents: Please contact the principal investigator for the supporting documents.
When available: With publication

Additional Information
Who can access the data: Please contact the principal investigator for the supporting documents.
Types of analyses: For any purpose.
Mechanisms of data availability: After approval of a proposal with a signed data usage agreement.
